# Choline Supplementation Alters Hippocampal Cytokine Levels in Adolescence and Adulthood in an Animal Model of Fetal Alcohol Spectrum Disorders

**DOI:** 10.3390/cells12040546

**Published:** 2023-02-08

**Authors:** Jessica A. Baker, Tamara S. Bodnar, Kristen R. Breit, Joanne Weinberg, Jennifer D. Thomas

**Affiliations:** 1 Center for Behavioral Teratology, San Diego State University, San Diego, CA 92120, USA; 2 Department of Cellular and Physiological Sciences, Faculty of Medicine, University of British Columbia, Vancouver, BC V6T 1Z3, Canada; 3 Department of Psychology, West Chester University, West Chester, PA 19383, USA

**Keywords:** ethanol, alcohol, choline, cytokine, neuroimmune, hippocampus, FASD

## Abstract

Alcohol (ethanol) exposure during pregnancy can adversely affect development, with long-lasting consequences that include neuroimmune, cognitive, and behavioral dysfunction. Alcohol-induced alterations in cytokine levels in the hippocampus may contribute to abnormal cognitive and behavioral outcomes in individuals with fetal alcohol spectrum disorders (FASD). Nutritional intervention with the essential nutrient choline can improve hippocampal-dependent behavioral impairments and may also influence neuroimmune function. Thus, we examined the effects of choline supplementation on hippocampal cytokine levels in adolescent and adult rats exposed to alcohol early in development. From postnatal day (PD) 4–9 (third trimester-equivalent), Sprague–Dawley rat pups received ethanol (5.25 g/kg/day) or sham intubations and were treated with choline chloride (100 mg/kg/day) or saline from PD 10–30; hippocampi were collected at PD 35 or PD 60. Age-specific ethanol-induced increases in interferon gamma (IFN-γ), tumor necrosis factor alpha (TNF-α), and keratinocyte chemoattractant/human growth-regulated oncogene (KC/GRO) were identified in adulthood, but not adolescence, whereas persistent ethanol-induced increases of interleukin-6 (IL-6) levels were present at both ages. Interestingly, choline supplementation reduced age-related changes in interleukin-1 beta (IL-1β) and interleukin-5 (IL-5) as well as mitigating the long-lasting increase in IFN-γ in ethanol-exposed adults. Moreover, choline influenced inflammatory tone by modulating ratios of pro- to -anti-inflammatory cytokines. These results suggest that ethanol-induced changes in hippocampal cytokine levels are more evident during adulthood than adolescence, and that choline can mitigate some effects of ethanol exposure on long-lasting inflammatory tone.

## 1. Introduction

According to the Centers for Disease Control and Prevention (CDC), 1 in 13 women report drinking alcohol during pregnancy [1,2]. This is concerning, as alcohol can cross the placenta to the fetus and lead to developmental disturbances referred to as fetal alcohol spectrum disorders (FASD) [3]. In the United States, the prevalence of FASD is estimated at approximately 2–7% [4,5,6]. Individuals with FASD display a wide range of physical, neuropathological, cognitive, and behavioral alterations that present in childhood and can persist throughout life [7]. Among the adverse effects associated with prenatal alcohol exposure are long-term alterations of the neuroimmune system, including abnormal microglial activity and dysregulation of cytokine levels (as reviewed in [8,9]). Importantly, microglia and cytokines are involved in a range of diverse functions outside of the immune response, including proper brain development that extends from the prenatal period through adolescence [10]. Ethanol-induced alterations in neuroimmune function during these vulnerable times may contribute to FASD pathology [8,9]. In fact, other neurodevelopmental disorders have been linked to abnormal immune mechanisms during pregnancy or during early childhood, such as autism spectrum disorders [11,12] and schizophrenia [13].

It is well known that cytokine levels, in both the central and peripheral immune system, are influenced by alcohol exposure [14]. Although ethanol and immune/neuroimmune interactions have been extensively investigated in adult alcohol exposure paradigms [14], and more recently during adolescence [15], less is known about the effects of fetal alcohol exposure on immune/neuroimmune function. An initial clinical study examined peripheral cytokines from chronic alcohol users during pregnancy and found elevated pro-inflammatory cytokines, such as IL-6 and TNF-α in both maternal and fetal blood samples [16]. More recent clinical studies show similar findings in maternal blood samples that are correlated with neurobehavioral impairments in infants [17]. Likewise, cytokine dysregulation has been reported in young children, including IL-10 and IFN-γ, which are associated with alcohol exposure during development and adverse child development [18,19]. Although results from clinical studies indicate that prenatal alcohol exposure can influence peripheral cytokine levels, it is difficult to examine neuroimmune responses in clinical studies.

Animal models of FASD have provided a valuable tool to examine neuroimmune changes after alcohol exposure during development. Multiple studies have reported prenatal ethanol-induced cytokine dysregulation in several brain regions, such as the hippocampus [8,9]. Evidence of prenatal ethanol-related neuroimmune alterations have been identified in neonates, as well as adults [8,9]. For example, ethanol-induced alterations in hippocampal cytokine levels, including TNF-α, IFN-γ, and IL-5, have been found in neonatal subjects [20,21,22,23,24,25]. Similar cytokine changes in the hippocampus and other brain regions have been identified in adult subjects exposed to alcohol during early development [26,27,28,29]. However, limited studies have examined neuroimmune alterations in adolescent subjects exposed prenatally to alcohol, although those that have found ethanol-related gene expression changes in cytokines that were distinct from those found later in adulthood [30,31]. Collectively, preclinical and clinical studies suggest that prenatal alcohol exposure can have age-specific effects on immune and neuroimmune functions, particularly cytokine dysregulation. Elucidation of these ethanol-induced neuroimmune changes may help us better understand FASD neuropathology and behavioral impairments.

Cytokine dysregulation may also be an important target for interventions that can improve outcomes following prenatal alcohol exposure. A number of interventions for FASD are currently being investigated, including supplementation with the essential nutrient choline. Choline is found in a variety of food products and plays a critical role throughout life, particularly during pre- and early postnatal development [32,33]. Animal studies have illustrated that perinatal choline supplementation can lead to long-lasting improvement in cognitive outcomes, even reducing age-related impairments in cognition [34,35]. Choline can also modify the effects of alcohol on the developing fetus. For example, preclinical studies have demonstrated that choline deficiency can exacerbate the teratogenic effects of developmental ethanol exposure [36]. In contrast, choline supplementation either during or after developmental alcohol exposure mitigates ethanol’s effects on development [37,38,39,40]. In particular, early postnatal choline treatment improves performance on tasks that depend on the integrity of the hippocampus and/or prefrontal cortex, suggesting that these brain areas that are targeted by prenatal ethanol exposure are amenable to choline-induced improvement in function [41,42,43]. Preclinical studies are supported by recent clinical data [44,45]; in fact, one clinical trial of choline supplementation in toddlers with FASD found long-lasting improvements in memory and attention [46].

The mechanisms by which choline exerts beneficial effects, however, are not well understood. Choline is a precursor to the neurotransmitter acetylcholine, acts as a methyl donor, serves as a precursor to membrane constituents such as phospholipids and sphingomyelin, and affects lipid metabolism [47]. All of these actions can lead to long-lasting changes in neuronal development. Interestingly, there are also interactions between choline and immune cells. In fact, there is evidence that choline works through anti-inflammatory mechanisms in both neurodegenerative [48,49] and neurodevelopmental [50] models. Choline can act on alpha-7 nicotinic acetylcholine (α7nACh) receptors to directly alter microglial cytokine production and release [51,52] in many brain regions, including the hippocampus, which can modulate memory and plasticity [49]. Similarly, another study found administration of choline increased IL-10, an anti-inflammatory cytokine, and improved hippocampal-dependent contextual fear response in a postoperative animal model of neuroinflammation [53]. Moreover, choline supplementation also reduces neuroinflammatory markers, including TNF-α, in the hippocampus of mice after a lipopolysaccharide (LPS) immune challenge, leading to improvement in immune-induced cognitive and behavioral impairments [54].

Prenatal choline levels have also been shown to modulate immune responses. For example, placental cells exposed to inadequate amounts of choline show elevated levels of cytokines, such as IL-6 [55]. In contrast, choline supplementation during pregnancy reduced placental levels of a number of immune markers, including IL-1β and TNF-α [56]. In addition, choline supplementation during gestation was shown to reduce LPS-induced immune responses in maternal serum and placental cells [57]. Thus, taken together, choline could have beneficial effects on behavioral and cognitive outcomes in individuals with FASD by modifying ethanol-induced immune dysregulation.

Although studies have shown that prenatal ethanol can have both short-term effects on the developing neonatal brain, as well as long-term effects in the mature adult brain, less is known about effects on the adolescent brain. This is extremely important as the neuroimmune system is still maturing during adolescence and alcohol insults early in perinatal development can impact normal adolescent brain development, which relies heavily on neuroimmune activity via cytokine signaling [10]. Moreover, given that adolescence marks a vulnerable time for brain development, this developmental period could therefore be a pivotal window for treatment and interventions to reduce long-term effects of early prenatal insults. We recently found that perinatal choline may modify cytokine responses to an LPS challenge in adults [58]. Therefore, the purpose of the current study was to (1) examine the effects of ethanol exposure during development on cytokine levels in the adolescent and adult hippocampus and (2) investigate the ability of choline supplementation, during early postnatal and adolescence period, to modify ethanol-induced cytokine alterations at either age.

## 2. Materials and Methods

### 2.1. Subjects

All study procedures were approved by the San Diego State University (SDSU) Institutional Animal Care and Use Committee (IACUC) and are in accordance with the National Institute of Health’s *Guide for the Care and Use of Laboratory Animals*. Male and female Sprague–Dawley rats were obtained from Charles River Laboratories (Hollister, CA, USA) on postnatal day (PD) 60 and allowed to acclimate for at least 2 weeks prior to breeding at the Center for Behavioral Teratology (CBT) at SDSU. One male and one female were paired together overnight, and the presence of a seminal plug indicated pregnancy; this day was identified as gestational day (GD) 0. On GD 0, pregnant dams were singly housed in standard cages on a 12:12 h light–dark cycle in temperature- and humidity-controlled rooms with access to food and water ad libitum. The day of birth (usually GD 22) was designated postnatal day (PD) 0; on PD 1 litters were culled to 8 pups with 4 males and 4 females (whenever possible). On PD 7, the paw pad of the pups was tattooed with non-toxic veterinary black ink (STONE Manufacturing and Supply Company, Kansas City, MO, USA) diluted with saline for identification purposes and to keep investigators blind to treatment condition. Subjects were weaned on PD 21 and separated by sex on PD 28. Subjects used in the present study were all bred in the vivarium at the CBT at SDSU.

### 2.2. Study Design

**Figure 1:**
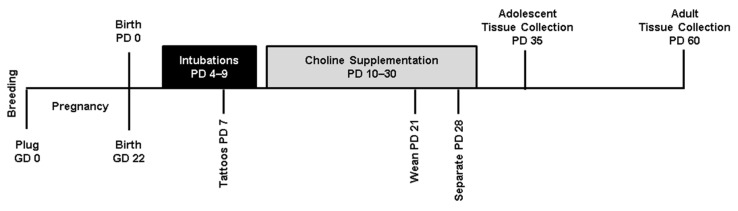
Timeline of experimental procedures. GD: gestational day, PD: postnatal day.

On PD 4, subjects were randomly assigned to experimental groups. To control for potential litter effects, only one sex pair (one male and one female) per treatment group was used from each litter (total litters used = 25). Subjects received either developmental ethanol (EtOH) exposure via intragastric intubation or sham intubations from PD 4–9, as well as either postnatal choline supplementation or saline from PD 10–30 (Figure 1). Subjects were sacrificed on PD 35 (adolescence) or PD 60 (adulthood) to assess both acute and long-term effects of developmental ethanol exposure and choline supplementation. Thus, this study utilized 8 exposure groups in a 2 (EtOH, sham) × 2 (choline, vehicle) × 2 (adolescent, adult) design.

### 2.3. Developmental Ethanol Exposure

Postnatal EtOH exposure occurred daily from PD 4–9, a period that mimics the “brain growth spurt” during the human 3rd trimester [59]. EtOH (95%; Sigma-Aldrich, St. Louis, MO, USA; 11.9% *v*/*v*; 5.25 g/kg/day) was added to an artificial milk diet [60] and was administered via intragastric intubation (PE-10 tubing; BrainTree Scientific, Inc., Braintree, MA, USA) to model binge exposure [61]. These dose and alcohol exposure parameters are based on the extensive literature [21,23,37,38,42,62,63,64,65,66,67,68,69] and utilized according to this modelling, as it is known to produce deficits in hippocampal-based tasks [37,38,42,65,66,67]. The EtOH-milk diet solution was intubated (27.5 mL/kg) twice per day, 2 h apart. Because intoxicated neonates may not adequately suckle, 2 additional milk-only feedings were provided (2 h apart). Subjects not assigned to receive developmental EtOH exposure received 4 sham intubations, but were not administered EtOH or milk [61,70]. Binge-like alcohol exposure during this developmental period leads to a transient lag in body growth after the first day of alcohol exposure [71,72,73], with eventual catch-up. We evaluated offspring growth in the present study and, similar to previous findings, we found significant effects of ethanol exposure on offspring growth (*p* < 0.001).

### 2.4. Choline Supplementation

Subjects were injected daily with choline (100 mg/kg/day) or isovolumetric saline vehicle via subcutaneous (s.c.) injections from PD 10–30, a period equivalent to early infancy and childhood in humans [74]. Choline chloride (BalChem, New Hampton, NY, USA) was mixed with sterile saline (0.85% sodium chloride solution; Sigma-Aldrich, St. Louis, MO, USA), at a dose previously shown to be the most effective at reducing behavioral alterations associated with developmental EtOH exposure [38] . Subjects not assigned to receive choline supplementation received isovolumetric saline vehicle. Previous studies have not reported effects of postnatal choline supplementation on offspring body growth [37,38,42,60,72,73]. Similarly, the present study found no effects of choline supplementation on offspring growth (*p* = 0.220).

### 2.5. Tissue Collection

Half of all subjects in each group were euthanized via carbon dioxide on PD 35. Brains were extracted and the hippocampus rapidly dissected on ice from each subject. Hippocampi were immediately flash frozen in liquid nitrogen and stored at −80 °C. The remaining subjects were euthanized on PD 60 using the same tissue collection protocol. Hippocampi were shipped on dry ice to the University of British Columbia for further processing.

### 2.6. Tissue Homogenization

Lysis buffer was prepared containing 150 mM NaCl, 20 mM Tris pH 7.5, 1 mM EDTA, 1 mM EGTA, and 1% Triton X-100, and immediately prior to homogenization the following were added (per 10 mL lysis buffer): 1 complete mini protease inhibitor cocktail tablet (Roche Diagnostics, Indianapolis, IN, USA), 100 µL phosphate inhibitor 2 and 3 (Sigma-Aldrich, St. Louis, MO, USA), 100 µL 1 M NaF, and 40 µL PMSF (from 500 mM stock in DMSO). Hippocampal samples were added to 1.6 mL tubes containing 8 zirconium oxide beads and 200 µL lysis buffer. Samples were homogenized using the Omni Bead Ruptor 24 (Omni International, Kennesaw, GA, USA) in 4 cycles (speed: 2.10, time: 5 s), with 1 min on ice in between cycles. Following homogenization, tissue samples were centrifuged at 1400× *g* for 10 min at 4 °C. Separate aliquots of supernatant were removed for protein quantification and cytokine analysis and stored at −20 °C until assayed.

### 2.7. Protein Measurements

Total protein levels were quantified in tissue homogenates using the Pierce Microplate BCA Protein Assay Kit (reduction agent compatible; Pierce Biotechnology, Rockford, IL, USA). Tissue homogenates were diluted (1:41) and the standard BCA protocol was followed with one modification: 5 µL (instead of the standard 4 µL) of compatibility reagent solution was added to each sample. Tissue homogenate samples were run in quadruplicate and the average protein concentration (µg/mL) across the 4 samples was calculated (samples with CVs ≥ 10 were re-run). Tissue cytokine levels were then adjusted, and values reported as pg cytokine/mg of protein.

### 2.8. Cytokine Measurements

Multiplex cytokine assays were performed using the Meso Scale Discovery (MSD) proinflammatory panel 1 rat V-PLEX kit (catalog #: K15059D, MSD, Rockville, MD, USA). This 9-plex cytokine panel allows for the simultaneous measurement of interleukin-1 beta (IL-1β), interleukin-4 (IL-4), interleukin-5 (IL-5), interleukin-6 (IL-6), interleukin-10 (IL-10), interleukin-13 (IL-13), interferon gamma (IFN-ɣ), keratinocyte chemoattractant/human growth-regulated oncogene (KC/GRO, a.k.a.CXCL1), and tumor necrosis factor (TNF-α). Samples were diluted in diluent 42 (1:2 dilution) and assays were performed using the standard MSD protocol. Cytokine plates were read using a Sector Imager 2400 (MSD, Rockville, MD, USA) and data analyzed using the MSD Discovery Workbench software v. 4.0 (MSD, Rockville, MD, USA). The lower limit of detection (LLOD) for the assays varied by analyte. The following LLODs were observed (pg/mg)—IL-1β: 9.58; IL-4: 0.78; IL-5: 12.30; IL-6: 52.90; IL-10: 2.37; IL-13: 0.99; IFN-ɣ: 1.26; TNF-α: 0.75; KC/GRO: 0.86. Cytokines falling below the LLOD were assigned a value of 0.

### 2.9. Statistical Analyses

Overall cytokine profiles across groups were analyzed using gplots package (version 3.1.3) in R software (version 4.2, R Foundation for Statistical Computing, Vienna, Austria) and visualized using heatmaps (GraphPad, Prism 8, San Diego, CA, USA) and dendrograms (produced in R, visualized using BioRender.com). Dependent variables were cytokine levels (pg/mg), cytokine difference scores (adolescent cytokine average per group subtracted form adult cytokine levels) [58], and pro-to-anti-inflammatory ratios [17].

Data were analyzed using the Statistical Packages for the Social Sciences (SPSS Version 27; IBM) with significance levels of *p* < 0.05 and trend towards significance at *p* < 0.10. Outliers were identified by z-scores (>|3.29|) and were Winsorized prior to analysis. Cytokines that were non-normally distributed were Blom transformed for statistical analyses. IL-4 levels were undetectable in the majority of the samples, and were excluded from further analysis, with the exception of the heatmaps and dendrograms. Cytokine levels were analyzed by ANOVAs in a 2 (EtOH: EtOH, sham) × 2 (Choline: choline, saline) × 2 (Age: adolescent, age) design. Initial analysis included sex as an additional factor; however, there were no significant sex effects or interactions, and thus sexes were collapsed and removed as a factor. Nevertheless, Supplemental Table S1 shows the cytokine levels for each group separated by sex for reference purposes. Post hoc analyses were conducted using Fisher’s Least Significant Difference (LSD) Tests. Data that did not pass normality tests were analyzed using non-parametric analyses; these examined the main effects of Ethanol, Choline, and Age (Mann–Whitney U) or group effects (Kruskal–Wallis H), with follow-up comparisons analyzed by chi-square. Means (M) and standard errors of the mean (SEM) are shown in graphs. All graphs illustrate the Winsorized, untransformed data.

## 3. Results

### 3.1. Overall Cytokine Profiles

**Figure 2:**
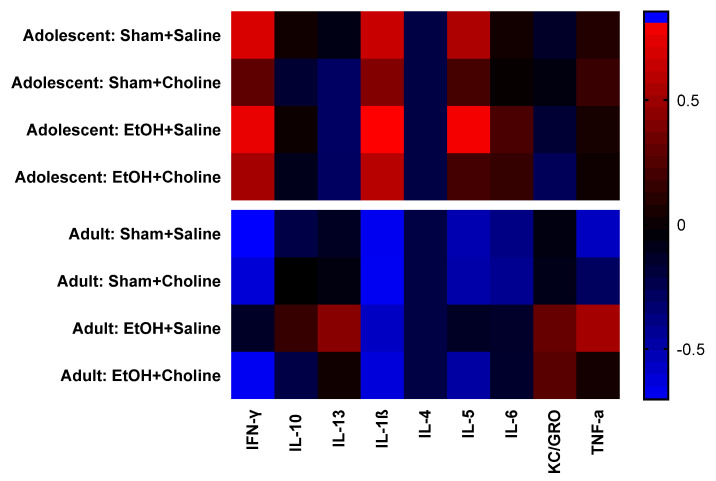
Hippocampal cytokine profiles during adolescence and adulthood. Overall, analysis demonstrates a noticeable clustering of cytokines patterns by age. Rows represent groups, as indicated, and the columns represent mean cytokine levels (*z*-scored data) for each group. Colors demonstrate deviations from the mean of zero, as indicated in the color key. EtOH: Ethanol.

**Figure 3:**
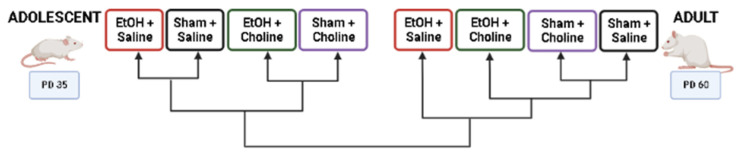
Dendrogram of treatment groups during adolescence or adulthood based on overall hippocampal cytokine profiles. In adolescence, choline supplemented groups clustered together as did non-supplemented groups, regardless of ethanol exposure. In contrast, ethanol-exposed subjects differed the most from all other groups in adulthood. EtOH: Ethanol. Created using BioRender.com.

**Figure 4:**
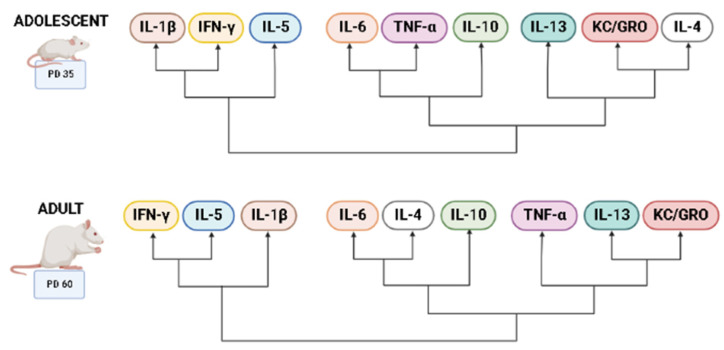
Dendrograms of cytokine grouping during adolescence and adulthood. Cytokine clustering shows similarities in adolescence and adulthood, despite differential cluster of groups within each age. Created using BioRender.com.

Heatmaps were used to visualize overall cytokine patterns in adolescents and adults across the four treatment groups (Figure 2). There was a striking age difference in hippocampal cytokine profiles, with higher average cytokine levels in adolescent compared to adult subjects. This was also evident in the hierarchical clustering analysis of cytokine profiles, with age emerging as the highest level of grouping (Figure 3). Interestingly, clustering analyses showed differential grouping of treatments within each age. During adolescence, cytokine profiles were more similar between choline supplemented subjects compared to non-supplemented subjects, regardless of ethanol exposure. In contrast, during adulthood, cytokine profiles in ethanol-exposed subjects not treated with choline were most distinct compared to all other groups. Despite differences in the group comparisons across age, the cytokine clustering analysis show similar grouping of cytokines, implying similar patterns of cytokines, in both adolescent and adult subjects, with only subtle differences detected (Figure 4).

### 3.2. Cytokine Levels

**Figure 5:**
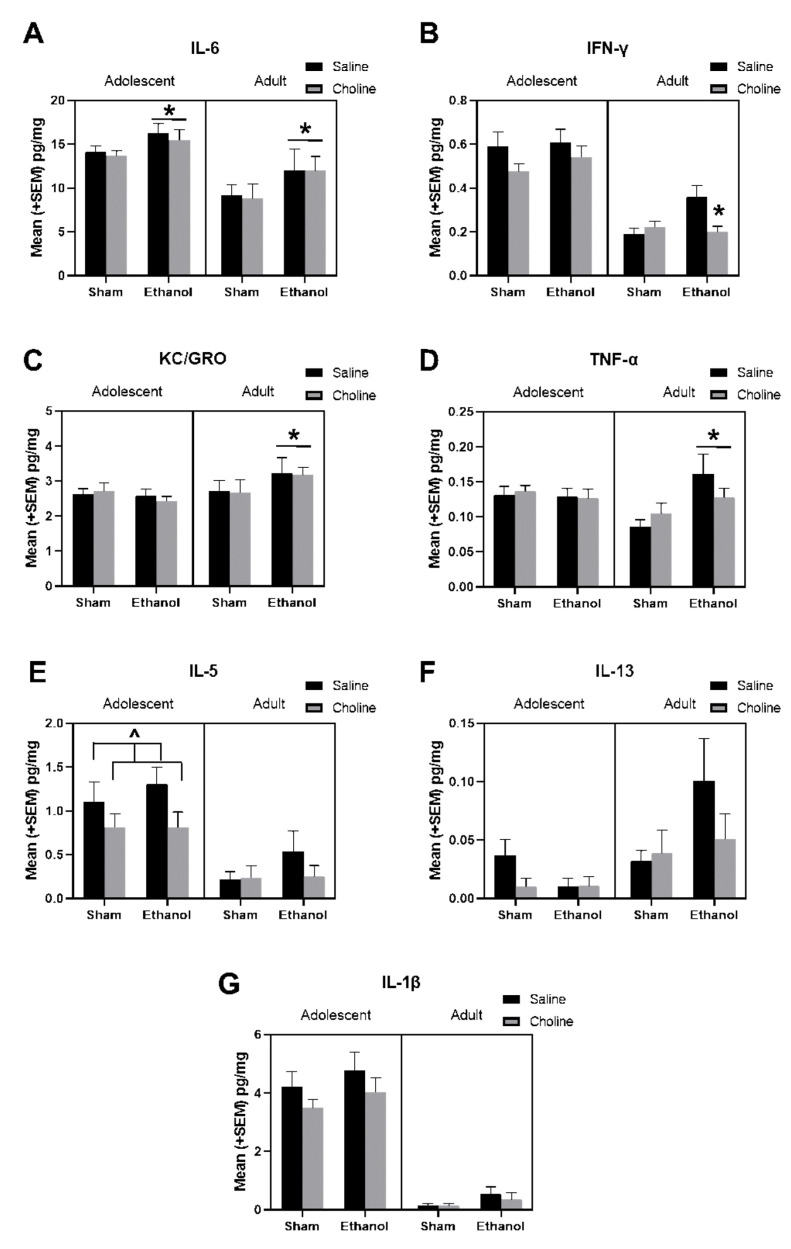
Developmental alcohol exposure and choline supplementation altered levels at cytokines during adolescence and adulthood. The following hippocampal cytokines at adolescence and adulthood are depicted in the graphs above: (**A**) IL-6 (EtOH > Sham, * *p* < 0.01; Adolescent > Adult, *p* < 0.01), (**B**) IFN-γ (EtOH + Choline < EtOH + Saline, * *p* < 0.05; Adolescent > Adult, *p* < 0.001), (**C**) KC/GRO (Adult: EtOH > Saline, * *p* < 0.05), (**D**) TNF-α (Adult: EtOH > Saline, * *p* < 0.05), (**E**) IL-5 (Adolescent: Choline < Saline, ^ *p* = 0.07; Adolescent > Adult, *p* < 0.001), (**F**) IL-13 (Choline < Saline, *p* = 0.12; Adolescent < Adult, *p* < 0.05), and (**G**) IL-1β (Adolescent > Adult, *p* < 0.001). n = 18–24 per group.

As expected, based on the heatmap, almost all individual cytokine levels significantly differed by age (Figure 5). Most cytokines declined with age, including IL-6 (U = 1735, *p* < 0.01), IFN-γ (*F*_(1,161)_ = 120.09, *p* < 0.001), TNF-α (*F*_(1,161)_ = 4.08, *p* < 0.05), IL-5 (U = 1653, *p* < 0.001), and IL-1β (U = 129, *p* < 0.001, Figure 5). In contrast, IL-13 (U = 2692, *p* < 0.05) significantly increased from adolescence to adulthood.

Subjects exposed to ethanol, regardless of age, had significantly higher levels of IL-6 (U = 2555, *p* < 0.01, Figure 5A). There were significant interactions of EtOH*Age for levels of KC/GRO (*F*_(1,161)_ = 3.66, *p* < 0.05, Figure 5C) and TNF-α (*F*_(1,161)_ = 5.37, *p* < 0.05, Figure 5D), as ethanol increased both cytokines in adults, but not adolescents. Interestingly, analysis of IFN-γ revealed a significant main effect of ethanol (*F*_(1,161)_ = 3.78, *p* < 0.05), as well as a 3-way interaction of Age*EtOH*Choline (*F*(_1,161)_ = 3.85, *p* < 0.05) Figure 5B). Ethanol increased IFN-γ in adult, but not adolescent subjects, and choline supplementation mitigated these long-lasting ethanol-related increases. In addition, as seen in Figure 5B, choline dampened IFN-γ levels overall, producing a main effect of choline (*F*_(1,161)_ = 4.16, *p* < 0.05). Similarly, although effects failed to reach statistical significance, it is notable that IL-5 was reduced in choline-treated subjects (U = 2880, *p* = 0.14, Figure 5E), particularly among adolescents (U = 534, *p* < 0.07). A similar, but not significant, pattern of choline-related reductions was observed in IL-13 (U = 2924, *p* = 0.12, Figure 5F).

### 3.3. Change in Cytokine Levels across Age

**Figure 6:**
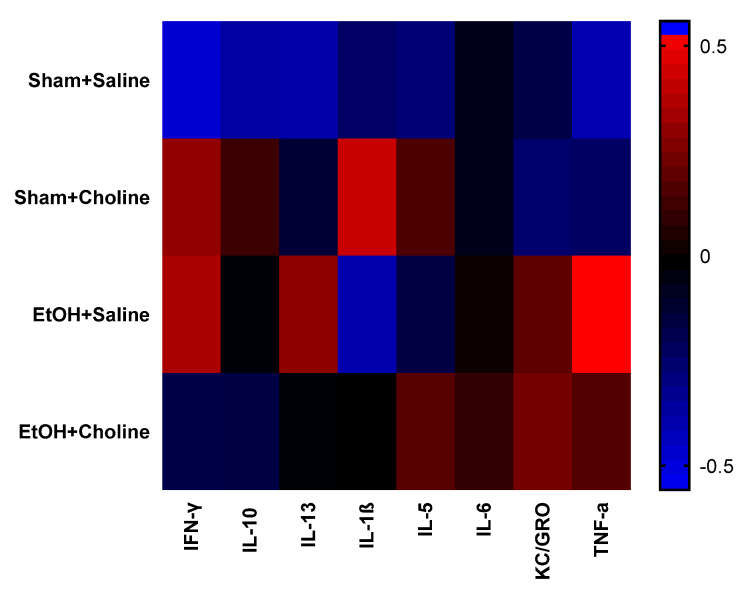
Change in hippocampal cytokine profiles across ages in each treatment group. Heatmaps depicting overall cytokine profiles the difference scores of adult cytokine levels compared to adolescent cytokines levels. Rows represent groups, as indicated, and the columns represent mean cytokine levels (z-scored data) for each group. Colors demonstrate deviations from the mean of zero, as indicated in the color key. EtOH: Ethanol.

Difference scores were calculated to investigate the effects of ethanol exposure and choline supplementation on cytokine levels across the two ages. First, heatmaps were used to assess differential developmental cytokine patterns across treatment groups (Figure 6), illustrating noticeable differences related to treatment. As seen, profiles of all treatment groups were different from that of sham + saline controls, although overall clustering analysis of treatment groups (data not shown) showed more similarities between ethanol-exposed subjects compared to non-ethanol-exposed subjects.

**Figure 7:**
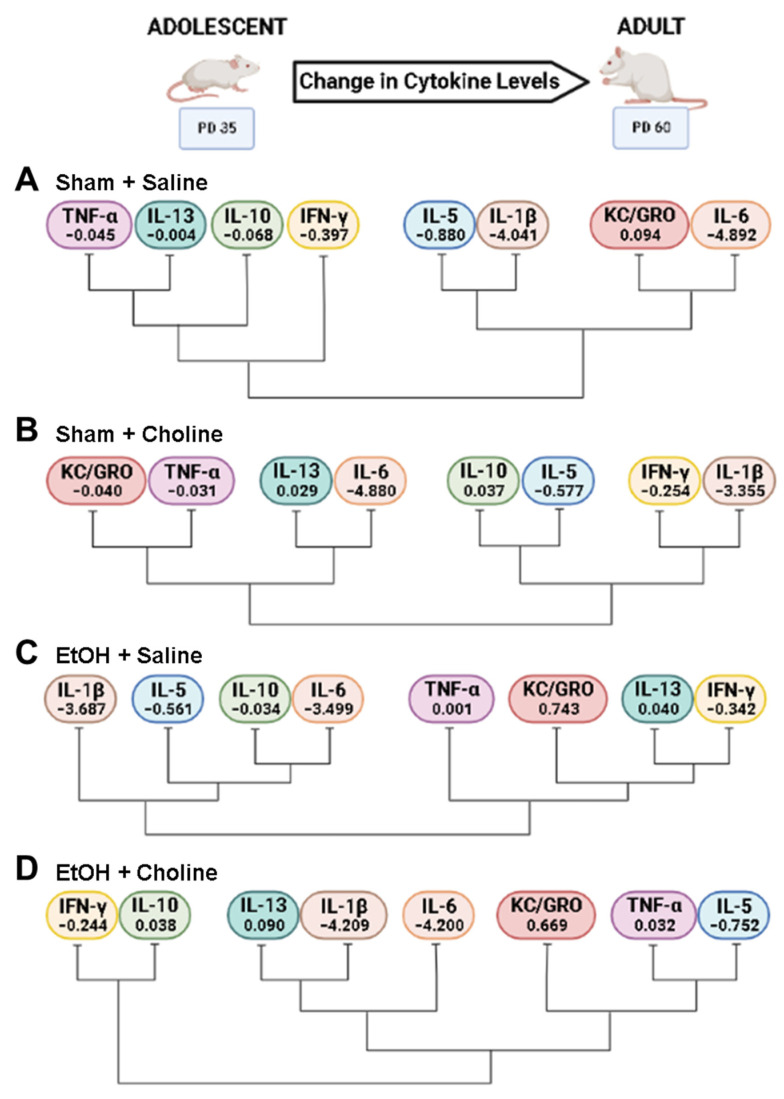
Clustering of cytokines across development in each treatment group. Differential clustering of cytokine changes across development are depicted in the treatment groups: (**A**) Sham + Saline, (**B**) Sham + Choline, (**C**) EtOH + Saline, (**D**) EtOH + Choline. The mean difference score for each cytokine within each group is depicted with each cytokine symbol. Negative numbers represent decrease in cytokine levels from adolescent to adulthood, whereas positive numbers show an increase. EtOH: Ethanol. Created using BioRender.com.

Because there were discernible age-related differences among groups, we next evaluated cytokine clustering across ages. Unique clustering of cytokines were identified for each treatment group (Figure 7), specifically for pro-inflammatory (IFN-γ, IL-1β, KC/GRO, TNF-α) and anti-inflammatory cytokines (IL-5, IL-10, IL-13). Unlike overall cytokine clustering based on age, where the relationship among cytokines remained similar (Figure 4), we see discernable patterns of cytokine changes that differ based upon both ethanol exposure and choline supplementation, so each treatment group exhibits unique cytokine changes over time.

**Figure 8:**
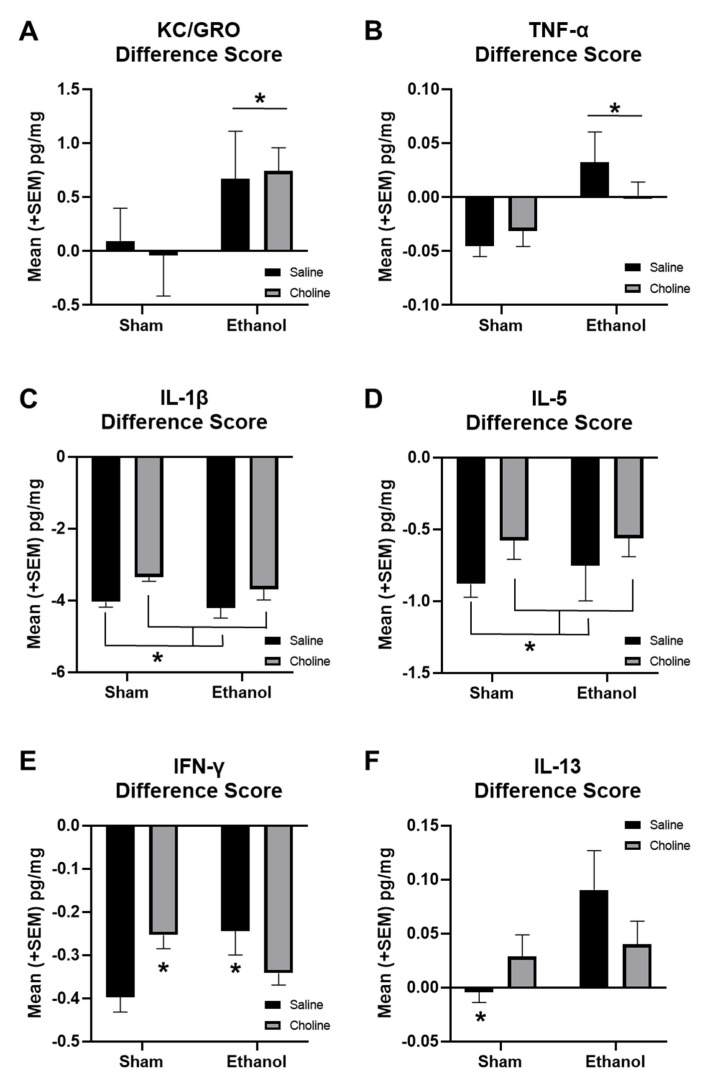
Developmental EtOH exposure and choline supplementation alter cytokine levels across development. Difference scores from adolescent to adulthood are used to show change in cytokine levels across development for (**A**) KCGRO (EtOH > Sham, * *p* < 0.05), (**B**) TNF-α (EtOH > Sham, * *p* < 0.01), (**C**) IL-1β (Choline < Saline, * *p* < 0.001), (**D**) IL-5 (Choline < Saline, * *p* < 0.001), (**E**) IFN-γ (Sham + Saline < EtOH + Saline & Sham + Choline * *p*’s < 0.05), and (**F**) IL-13 (Sham + Saline < all other groups, * *p’*s < 0.01). n = 19–24 per group.

To further examine changes across age, difference scores (adult–adolescent) for individual cytokines were calculated (Figure 8). Cytokines that showed significant main effects of ethanol include KC/GRO (*F*_(1,86)_ = 4.39, *p* < 0.05, Figure 8A) and TNF-α (*F*_(1,86)_ = 9.05, *p* < 0.01, Figure 8B). KC/GRO increased with age in ethanol-exposed, but not sham, subjects. In contrast, TNF-α declined with age among sham but not ethanol-exposed subjects, an effect particularly driven by non-supplemented subjects. Choline supplementation reduced age-related changes in some cytokines, including IL-1β (*F*_(1,86)_ = 14.41, *p* < 0.001, Figure 8C) and IL-5 (*F*_(1,86)_ = 23.41, *p* < 0.001, Figure 8D). Overall, cytokine levels decreased from adolescence to adulthood. However, this reduction was blunted in choline-supplemented subjects, likely due to general reductions in adolescent levels of cytokines, specifically IFN-γ, IL-1β, and IL-5.

Interestingly, there were significant interactions between ethanol exposure and choline supplementation for changes in IFN-γ (*F*_(1,86)_ = 9.43 *p* < 0.01, Figure 8E) and IL-13 (*F*_(1,86)_ = 5.89, *p* < 0.05, Figure 8F) from adolescence to adulthood. For both cytokines, there were significant age-related differences between sham control and sham choline groups (*p*’s < 0.01), likely a result of lower IFN-γ and IL-13 levels in choline-treated subjects during adolescence. In addition, ethanol-exposed subjects not treated with choline also differed significantly from sham controls for both cytokines (*p*’s < 0.05). Developmental changes of IFN-γ in ethanol-exposed subjects treated with choline did not differ from that of sham controls.

### 3.4. Balance of Pro-Inflammatory to Anti-Inflammatory Cytokines across Development

**Figure 9:**
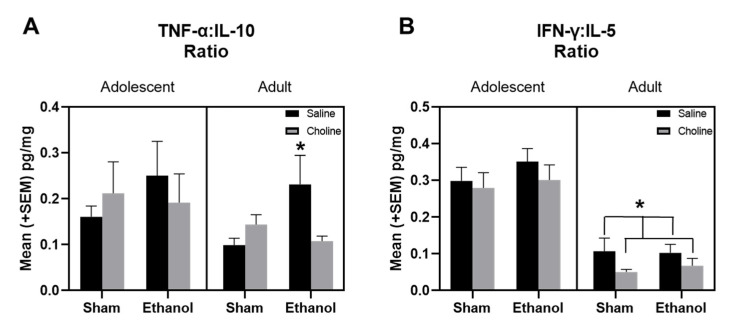
EtOH exposure and choline supplementation altered ratio of pro-inflammatory to anti-inflammatory cytokines. (**A**) Ratio of pro-inflammatory cytokine, TNF-α, to anti-inflammatory cytokine, IL-10 during adolescence and adulthood. EtOH exposure and choline supplementation altered the ratios of TNF-α:IL-10 in adults (EtOH + Saline > Sham + Saline, * *p* < 0.05), but not adolescents. (**B**) Ratio of pro-inflammatory cytokine, IFN-γ, to anti-inflammatory cytokine, IL-5 during adolescence and adulthood. Choline supplementation altered ratios of IFN-γ:IL-5 in adults (Choline < Saline, * *p* < 0.05) but not adolescents. n = 18–19 per group.

As we identified differential clustering of pro- and anti-inflammatory cytokines in our treatment groups across development, we examined ratios of pro-inflammatory cytokines, TNF-α and IFN-γ, to anti-inflammatory cytokines, IL-10 and IL-5, in adolescent and adult subjects (Figure 9). There was a significant effect of age in the ratios of both TNF-α:IL10 (*F*_(1,161)_ = 7.65, *p* < 0.01, Figure 9A) and IFN-γ:IL-5 (*F*_(1,161)_ = 95.51, *p* < 0.001, Figure 9B). Interestingly, there was a significant interaction between ethanol exposure and choline supplementation in the TNF-α:IL10 ratios in adult subjects (*F*_(1,161)_ = 5.80, *p* < 0.05), but not adolescents. In adults, choline supplementation significantly attenuated ethanol-induced increases in TNF-α:IL10 ratios and brought inflammatory tone back to ratio levels seen in non-exposed adults. Choline supplementation also reduced ratios of IFN-γ:IL-5 in adult subjects (*F*_(1,161)_ = 5.69, *p* < 0.05), regardless of ethanol exposure.

## 4. Discussion

The current study aimed to examine the effects of choline supplementation on cytokine levels in the hippocampus of both adolescent and adult-aged subjects exposed to alcohol during development. We found age-specific effects of ethanol exposure for many cytokines, including IFN-γ, TNF-α, and KC/GRO, with ethanol-related increases seen mostly in adulthood, as compared to adolescence. However, there was one exception, IL-6, that showed persistent ethanol-induced increases at both ages. Importantly, choline supplementation mitigated long-lasting ethanol-induced increases of IFN-γ in adult subjects. In addition, cytokine levels showed generally blunted patterns in choline-supplemented subjects, demonstrated by age-related changes (difference scores) and ratios of pro- to anti-inflammatory cytokines. These results demonstrate that developmental alcohol exposure has long-lasting effects on neuroimmune function that may not be evident during adolescence, and that choline supplementation shows promise as a potential intervention strategy in this preclinical model.

There was a clear age difference in hippocampal cytokine levels, with higher cytokines detected in adolescent compared to adult subjects. Adolescence represents a sensitive neurodevelopmental period that includes processes such as synaptogenesis and synaptic refinement, as well as neurotransmitter and interneuron maturation (as reviewed in [10]). The neuroimmune system also undergoes extensive maturation during this period and, along with neural activity, contributes to shaping brain development [10,75]. Although there has been very limited research on adolescent immune/neuroimmune function, the elevated levels of hippocampal cytokines detected here, in adolescence, as compared to adulthood, is likely indicative of microglial activation and cytokine release that is critical for adolescent brain development [10]. Importantly, however, despite heightened levels of hippocampal cytokines during adolescence, cytokine profiles (via hierarchical clustering; Figure 4) were strikingly similar between adolescent and adult subjects, suggesting functional parallels at the two ages.

During adolescence, most hippocampal cytokine levels were generally unaffected by early postnatal ethanol exposure. The exception was IL-6, which showed ethanol-induced elevations during adolescence that persisted into adulthood. These results are consistent with earlier reports that alcohol exposure during gestation increases central levels of IL-6 during early-life, albeit in the prefrontal cortex on postnatal day 8 [20] and in the whole embryo [76]. Although IL-6 has both pro- and anti-inflammatory effects, centrally, it has been shown to impact neuronal survival and differentiation, as well as modulate production of growth factors [77,78,79]. Of note, during early-life, heightened IL-6 levels have been shown to be associated with brain injury in preterm infants [80]. Moreover, in a mouse model, over-expression of IL-6 is associated with behavioral deficits, many of which are also known to occur following early exposure to alcohol, including impairments in cognitive function, learning, and social behavior, increased anxiety-like behaviors, and altered habituation via an imbalance of increased excitatory synapses and decreased inhibitory synapses [81]. Central administration of IL-6 has also been shown to induce depressive-like behavior in mice and importantly, this occurs in the absence of IL-1β changes, which would be more typical of sickness-related responses [82]. Indeed, in the current study, we did not find ethanol-induced alterations in IL-1β at either age. Finally, the finding that ethanol exposure increased IL-6 during both adolescence and adulthood suggests that this may be a long-lasting feature of developmental alcohol-exposure, and one that was not modified by choline supplementation.

Similar to IL-6, alterations in IL-5 were also detected in adolescent subjects. Specifically, in the current study, a trend for choline-induced reductions in the anti-inflammatory cytokine, IL-5, was detected in adolescent subjects, as well as in choline-related reductions in age-specific changes in IL-5. IL-5 is a Th2 cytokine that is produced by activated immune cells during allergic responses (reviewed in [83]), as well as in disease progression in asthma [84], and importantly, there is preliminary clinical evidence that there are increased rates of atopy (tendency to develop allergic conditions and associated with increased immune response to allergens) following prenatal alcohol exposure (reviewed in [85]). Interestingly, choline has been explored as a treatment option in allergic conditions. For example, in a model of allergic airway disease, subsequent choline administration was shown to dampen inflammatory responses, including IL-5, in bronchoalveolar lavage fluid [86]. Furthermore, in a human study of asthma, choline therapy also reduced peripheral IL-5 levels [87]. Although cytokine levels, such as IL-5, have traditionally been explored in peripheral samples and in bronchial fluids, there is growing evidence that immune system activation and, specifically, allergy induction impacts neuroimmune function, targeting the hippocampus [88]. As such, the trend for decreased hippocampal IL-5 following choline supplementation may reflect a widespread dampening of allergic-related immune profiles.

As previously stated, adolescence is a critical period for brain development mediated, at least in part, by immune function [10]. As such, and in line with our data, neuroimmune function is likely generally heightened during adolescence, as compared to adulthood when central maturational processes are complete. Thus, it is perhaps not surprising that the majority of the effects of ethanol and the modulatory effects of choline on hippocampal cytokines levels were detected in adult subjects, with potential masking of these effects in adolescence due to heightened neuroimmune function. For example, multiple cytokines showed ethanol-induced increases in adulthood but not adolescence, including TNF-α, KC/GRO, and IFN-γ. Changes in these cytokines are generally consistent with other studies of prenatal/developmental alcohol exposure. Specifically, hippocampal TNF-α has been shown to be increased on embryonic day 17 [26], postnatal day 8 [20], and in adulthood [89,90], whereas hippocampal IFN-γ was increased on postnatal day 8 [20] and hippocampal KC/GRO was increased on embryonic day 17 [26], in alcohol-exposed subjects compared to controls. Elevated TNF-α levels in the hippocampus appears to be the most consistent across prenatal/developmental alcohol exposure models and it has been reported that this may be a key driver of impaired synaptic plasticity in the hippocampus [90]. This is in line with findings from our group, which have identified deficits in hippocampal-based behaviours following developmental alcohol exposure [37,40,42,91]. Importantly, our data suggest that if one is examining the effects of prenatal alcohol on neuroimmune function among adolescents, they may miss important alcohol-related changes.

Importantly, in adults, choline supplementation dampened ethanol-induced increases in IFN-γ, reducing levels back to those found in control subjects. IFN-γ is an important cytokine and its primary signaling pathways are involved in functions such as the cell cycle, growth, and apoptosis [92]. In fact, IFN-γ has been found to regulate levels of multiple proteins [92] including numerous growth factors and cytokines, including TNF-α and IL-6 [93]. Moreover, IFN-γ is extremely important for proper function of the hippocampus, with altered IFN-γ levels linked to changes in neurogenesis, hippocampal plasticity, and learning and memory [93,94]. It is interesting that although the ethanol-induced effects on IFN-γ were not present until adulthood, there was a reduction in IFN-γ, albeit non-significant (*p* = 0.13), in choline-supplemented adolescent subjects. This suggests that although most ethanol-induced effects on cytokine levels do not appear until adulthood, choline’s influence on cytokine levels, such as IL-5 and IFN-γ might begin earlier in adolescence. As elevations in IFN-γ can also increase levels in other cytokines, including pro-inflammatory TNF-α and IL-6 [93], this choline-induced blunting of IFN-γ early in adolescence may alter neuroimmune responses and other developmental processes from adolescence through adulthood that may attenuate prenatal alcohol-induced cognitive and behavioral alterations.

Furthermore, it is noteworthy that the difference scores examining cytokine changes across age identified differential cytokine profiles based on both ethanol exposure and choline supplementation. Importantly, evaluating both adolescent and adult cytokine levels allowed for the developmental trajectory of neuroimmune activity to be assessed. Specifically, greater difference scores were detected in ethanol subjects than in controls for KC/GRO and TNF-α, driven by the unique pattern of elevated cytokines in ethanol-exposed adult subjects. In contrast, the difference scores for IL-1β and IL-5 were dampened with choline supplementation, as a result of lower levels of the cytokines that were present in adolescence. Dampening of adolescent cytokine levels also contributed to the unique developmental patterns detected for IFN-γ and IL-13. Taken together, these data indicate that cytokines vary in an age-dependent manner, which can be modified by both choline and developmental alcohol exposure.

Examination of changes in clustering of cytokines from adolescence to adulthood revealed unique cytokine patterns that differed depending on both ethanol exposure and choline supplementation. Therefore, we explored the relative balance/imbalance of pro- to anti-inflammatory cytokine signaling during both adolescence and adulthood by assessing ratios of relevant cytokines. Specifically, we examined the ratios of TNF-α:IL-10 [17] and IFN-ɣ:IL-5 [95,96] in order to generate two different, but complementary, assessments of hippocampal pro:anti-inflammatory cytokine balance. There were age-specific effects of both ratios with differences present in adults, but not adolescents. In the adults, there were ethanol-induced elevations in TNF-α:IL-10 ratios, suggestive of a pro-inflammatory shift. Interestingly, this cytokine imbalance between TNF-α:IL-10 has been previously linked with prenatal alcohol exposure, with increased ratios of maternal TNF-α:IL-10 associated with an increased risk of FASD in alcohol-exposed children as well as neurobehavioral impairments in infants with FASD [17]. More importantly, in the current study, these ethanol-induced alterations of TNF-α:IL-10 ratios in adults were mitigated by early choline supplementation, with levels similar to those of non-ethanol exposed controls. The consistency with clinical data provides further confidence in the importance of this ethanol-related cytokine imbalance and suggests that postnatal choline supplementation could be effective in altering inflammatory tone. This finding has important implications, as toddlers with FASD who have been treated with choline exhibit improved cognitive outcomes [97]. Although ethanol effects on IFN-γ:IL-5 ratios were not present in adolescent or adult subjects, there was a choline-induced decrease in the IFN-γ:IL-5 ratio in adults, suggestive of an anti-inflammatory shift. Interestingly, modifications of IFN-γ:IL-5 ratios have been identified in other studies examining early immune responses [95,96]. It is interesting to note that although altered pro:anti-inflammatory ratios have been identified early in development [17], similar to the current study, alterations in the ratios of TNF-α:IL-10 or IFN-γ:IL-5 have not been reported in adolescence, providing further evidence that adolescence is a distinct period for neuroimmune system development. Importantly, cytokines have been shown to act in concert with one another and function as a network, rather than individually, and our current results and previous research suggest that the balance between pro- and anti-inflammatory markers is crucial to neurodevelopmental outcomes [17,18,19,98].

Although the current study and others [53,54,58] have shown modulatory effects of choline supplementation on the neuroimmune system, the mechanisms by which choline exerts these long-term changes are not known. The pattern of reduced cytokines following early postnatal choline in both control and ethanol-exposed subjects suggests that choline has early effects on inflammatory tone. As noted above, choline can directly impact cytokine levels through acting on α7nACh receptors that are present on both microglia and neurons in many brain regions [51,52] including the hippocampus. This represents one possible mechanism by which choline may modify cytokines, as α7nACh receptors are present in hippocampal cells. Interestingly, expression of α7nACh receptors is age dependent with higher levels found in the fetal and neonatal hippocampus compared to adult levels [99]. Specifically, there is a transient peak between postnatal day 7–14 after which expression levels steadily decline to lower adulthood levels [99,100]. Thus in the current study, choline supplementation from postnatal 10–30 is occurring during a period of peak cholinergic receptor expression.

The results of the current study are important in that they examine the impact of developmental ethanol exposure and subsequent choline treatment at two key developmental periods, adolescence and adulthood. Unfortunately, there is a scarcity of data examining neuroimmune functioning during adolescence and more work is needed to better understand this sensitive period of development. In addition, the present study examined the hippocampus due to the well-established impacts of alcohol exposure on this brain region and subsequent behavioral alterations [101,102,103], as well as our previously reported work which highlighted the beneficial impact of choline supplementation on hippocampal-based tasks [40,43]. However, it will be important for future work to examine neuroimmune functioning in other brain areas affected by prenatal exposure to alcohol, such as the prefrontal cortex and cerebellum. In addition, the current study aimed to establish whether neuroimmune function could be dampened by choline supplementation following developmental alcohol exposure and it will be important that future work examine microglial activity to confirm that these key producers of central cytokines are, in fact, dampened. Finally, although our previous work has shown that choline supplementation improves behavior on hippocampal-based tasks, it will also be critical to examine both neuroimmune functioning and behavior function in the same subject, to directly link these two outcomes.

## 5. Conclusions

The present study evaluated the effects of ethanol exposure and choline supplementation during two critical developmental periods, adolescence and adulthood. Ethanol-induced changes in cytokine levels were more evident in the mature adult compared to the adolescent hippocampus. Although adolescence is thought to be a period of extended development during which the brain is still vulnerable to early life insults such as alcohol exposure, some have reported muted neuroimmune responses to immune challenges among adolescents but not adults exposed to alcohol [15]. The current results indicate that adolescence is characterized by elevations in hippocampal cytokines; it is possible that these elevations may mask the effects of developmental alcohol exposure. In contrast, patterns of blunted cytokine levels in choline-supplemented subjects were more evident during adolescence. Importantly, choline reduced the ratio of pro- to anti-inflammatory cytokines in adults, suggesting that choline alters long-term inflammatory tone. Thus, choline’s effects on neuroimmune function in the hippocampus could be a possible mechanism by which choline exerts beneficial effects on cognitive and behavioral outcomes in both preclinical and clinical models.

## Data Availability

The original contributions presented in the study are included in the article and Supplementary Materials. Further inquiries can be directed to the corresponding author (J.D.T.).

## Supplementary Materials

The following supporting information can be downloaded at: https://www.mdpi.com/article/10.3390/cells12040546/s1, Supplemental Table S1. Hippocampal cytokine levels in males and females.
