# Supplementary material for: Choline Supplementation Alters Hippocampal Cytokine Levels in Adolescence and Adulthood in an Animal Model of Fetal Alcohol Spectrum Disorders
Source: Cells. 2023 Feb 8;12(4):546. doi: 10.3390/cells12040546 (PMC9953782; doi:10.3390/cells12040546)
Supplement: Supplementary file 1 [file cells-12-00546-s001.zip › cells-2116856-supplementary.pdf]

Supplemental Table S1. Hippocampal Cytokine Levels in Males and Females

		Cytokine Levels (pg/mg; M ± SEM [n])																	
**Exposure Group**	**Sex**	**IL-4 Adolescent**	**IL-4 Adult**	**IL-5 Adolescent**	**IL-5 Adult**	**IL-6 Adolescent**	**IL-6 Adult**	**IL-10 Adolescent**	**IL-10 Adult**	**IL-13 Adolescent**	**IL-13 Adult**	**IFN-γ Adolescent**	**IFN-γ Adult**	**IL-1β Adolescent**	**IL-1β Adult**	**KC/GRO Adolescent**	**KC/GRO Adult**	**TNF-α Adolescent**	**TNF-α Adult**
Sham + Saline	Females:	0.00 ± 0.00 [n = 10]	0.00 ± 0.00 [n = 12]	1.24 ± 0.35 [n = 10]	0.25 ± 0.14 [n = 12]	15.08 ± 0.96 [n = 10]	7.27 ± 1.32 [n = 12]	0.08 ± 0.05 [n = 10]	0.00 ± 0.00 [n = 12]	0.05 ± 0.02 [n = 10]	0.03 ± 0.01 [n = 12]	0.67 ± 0.10 [n = 10]	0.16 ± 0.03 [n = 12]	4.83 ± 0.84 [n = 10]	0.16 ± 0.09 [n = 12]	2.74 ± 0.26 [n = 10]	3.28 ± 0.43 [n = 12]	0.13 ± 0.01 [n = 10]	0.08 ± 0.01 [n = 12]
	Males:	0.00 ± 0.00 [n = 9]	0.00 ± 0.00 [n = 12]	0.95 ± 0.30 [n = 9]	0.18 ± 0.12 [n = 12]	13.03 ± 0.98 [n = 9]	11.06 ± 1.92 [n = 12]	0.06 ± 0.06 [n = 9]	0.00 ± 0.00 [n = 12]	0.02 ± 0.02 [n = 9]	0.03 ± 0.01 [n = 12]	0.51 ± 0.07 [n = 9]	0.22 ± 0.04 [n = 12]	3.55 ± 0.55 [n = 9]	0.14 ± 0.10 [n = 12]	2.48 ± 0.21 [n = 9]	2.12 ± 0.41 [n = 12]	0.13 ± 0.02 [n = 9]	0.09 ± 0.02 [n = 12]
Sham + Choline	Females:	0.00 ± 0.00 [n = 10]	0.00 ± 0.00 [n = 12]	0.82 ± 0.23 [n = 10]	0.46 ± 0.24 [n = 12]	14.01 ± 0.88 [n = 10]	9.46 ± 2.18 [n = 12]	0.01 ± 0.01 [n = 10]	0.03 ± 0.03 [n = 12]	0.01 ± 0.01 [n = 10]	0.03 ± 0.02 [n = 12]	0.52 ± 0.06 [n = 10]	0.19 ± 0.02 [n = 12]	3.85 ± 0.48 [n = 10]	0.13 ± 0.13 [n = 12]	2.85 ± 0.43 [n = 10]	2.63 ± 0.49 [n = 12]	0.13 ± 0.01 [n = 10]	0.11 ± 0.02 [n = 12]
	Males:	0.00 ± 0.00 [n = 9]	0.00 ± 0.00 [n = 11]	0.81 ± 0.22 [n = 9]	0.00 ± 0.00 [n = 11]	13.32 ± 0.74 [n = 9]	8.08 ± 2.68 [n = 11]	0.02 ± 0.02 [n = 9]	0.08 ± 0.08 [n = 11]	0.01 ± 0.01 [n = 9]	0.05 ± 0.04 [n = 11]	0.43 ± 0.03 [n = 9]	0.26 ± 0.05 [n = 11]	3.10 ± 0.25 [n = 9]	0.14 ± 0.09 [n = 11]	2.56 ± 0.16 [n = 9]	2.72 ± 0.60 [n = 11]	0.15 ± 0.01 [n = 9]	0.10 ± 0.02 [n = 11]
EtOH + Saline	Females:	0.00 ± 0.00 [n = 10]	0.00 ± 0.00 [n = 10]	1.52 ± 0.27 [n = 10]	0.44 ± 0.29 [n = 10]	17.24 ± 1.77 [n = 10]	11.07 ± 3.05 [n = 10]	0.05 ± 0.05 [n = 10]	0.08 ± 0.08 [n = 10]	0.01 ± 0.01 [n = 10]	0.10 ± 0.05 [n = 10]	0.69 ± 0.09 [n = 10]	0.37 ± 0.08 [n = 10]	5.32 ± 0.87 [n = 10]	0.61 ± 0.39 [n = 10]	2.64 ± 0.23 [n = 10]	2.91 ± 0.57 [n = 10]	0.12 ± 0.02 [n = 10]	0.15 ± 0.04 [n = 10]
	Males:	0.00 ± 0.00 [n = 8]	0.00 ± 0.00 [n = 9]	1.03 ± 0.28 [n = 8]	0.65 ± 0.38 [n = 9]	15.11 ± 0.92 [n = 8]	13.10 ± 4.02 [n = 9]	0.08 ± 0.04 [n = 8]	0.13 ± 0.10 [n = 9]	0.01 ± 0.01 [n = 8]	0.10 ± 0.06 [n = 9]	0.51 ± 0.06 [n = 8]	0.35 ± 0.06 [n = 9]	4.10 ± 0.87 [n = 8]	0.45 ± 0.34 [n = 9]	2.48 ± 0.37 [n = 8]	3.60 ± 0.69 [n = 9]	0.14 ± 0.02 [n = 8]	0.18 ± 0.04 [n = 9]
EtOH + Choline	Females:	0.00 ± 0.00 [n = 10]	0.00 ± 0.00 [n = 11]	0.78 ± 0.31 [n = 10]	0.38 ± 0.22 [n = 11]	16.91 ± 2.11 [n = 10]	11.00 ± 1.52 [n = 11]	0.05 ± 0.05 [n = 10]	0.00 ± 0.00 [n = 11]	0.02 ± 0.01 [n = 10]	0.07 ± 0.04 [n = 11]	0.60 ± 0.09 [n = 10]	0.21 ± 0.03 [n = 11]	4.61 ± 0.83 [n = 10]	0.13 ± 0.13 [n = 11]	2.50 ± 0.21 [n = 10]	3.09 ± 0.32 [n = 11]	0.13 ± 0.01 [n = 10]	0.12 ± 0.01 [n = 11]
	Males:	0.00 ± 0.00 [n = 9]	0.00 ± 0.00 [n = 10]	0.85 ± 0.18 [n = 9]	0.11 ± 0.11 [n = 10]	13.89 ± 0.95 [n = 9]	13.04 ± 3.11 [n = 10]	0.02 ± 0.02 [n = 9]	0.00 ± 0.00 [n = 10]	0.00 ± 0.00 [n = 9]	0.03 ± 0.02 [n = 10]	0.48 ± 0.05 [n = 9]	0.19 ± 0.05 [n = 10]	3.40 ± 0.43 [n = 9]	0.58 ± 0.49 [n = 10]	2.36 ± 0.17 [n = 9]	3.27 ± 0.29 [n = 10]	0.13 ± 0.02 [n = 9]	0.13 ± 0.02 [n = 10]
